# Singly ionized double-donor complex in vertically coupled quantum dots

**DOI:** 10.1186/1556-276X-7-489

**Published:** 2012-08-31

**Authors:** Ramón Manjarres-García, Gene Elizabeth Escorcia-Salas, Ilia D Mikhailov, José Sierra-Ortega

**Affiliations:** 1 Group of Investigation in Condensed Matter Theory, Universidad del Magdalena, Santa Marta, Colombia; 2 Universidad Industrial de Santander, A. A. 678, Bucaramanga, Colombia

**Keywords:** Quantum dots, Adiabatic approximation, Artificial molecule, 78.67.-n, 78.67.Hc, 3.21.-b

## Abstract

The electronic states of a singly ionized on-axis double-donor complex (*D*_2_^+^) confined in two identical vertically coupled, axially symmetrical quantum dots in a threading magnetic field are calculated. The solutions of the Schrödinger equation are obtained by a variational separation of variables in the adiabatic limit. Numerical results are shown for bonding and antibonding lowest-lying artificial molecule states corresponding to different quantum dot morphologies, dimensions, separation between them, thicknesses of the wetting layers, and magnetic field strength.

## Background

Quantum dots (QDs) have opened the possibility to fabricate both artificial atoms and molecules with novel and fascinating optoelectronic properties which are not accessible in bulk semiconductor materials. An attractive route for nano-structuring semiconductor materials offers self-assembled quantum dots which are formed by the Stranski-Krastanow growth mode by depositing the material on a substrate with different lattice parameters [1-5]. The electrical and optical properties of these structures may be changed in a controlled form by doping the shallow impurities whose energy levels are defined by the interplay between the reductions of the physical dimension, the Coulomb attraction, and the inter-particle correlation.

Recently, it has been proposed to use the singly ionized double-donor system (*D*_2_^+^) confined in a single semiconductor QD [6] or ring [7] as an adequate functional part in a wide range of device applications, including spintronics, optoelectronics, photovoltaics, and quantum information technologies. This two-level system encodes logical information either on the spin or on the charge degrees of freedom of the single electron and allows us to manipulate conveniently its molecular properties, such as the energy splitting between the bonding and antibonding lowest-lying molecular-like states or the spatial distribution of carriers in the system [8-12]. One can expect that the singly ionized double-donor system (*D*_2_^+^) confined in vertically coupled QDs should have similar properties. In this paper, we analyze the electronic states of an artificial hydrogen molecular ion (*D*_2_^+^) compound by two positive ions that interchange their electron, which is constrained to exchange between two identical vertically coupled, axially symmetrical QDs in the presence of a threading magnetic field.

## Methods

**Figure 1:**
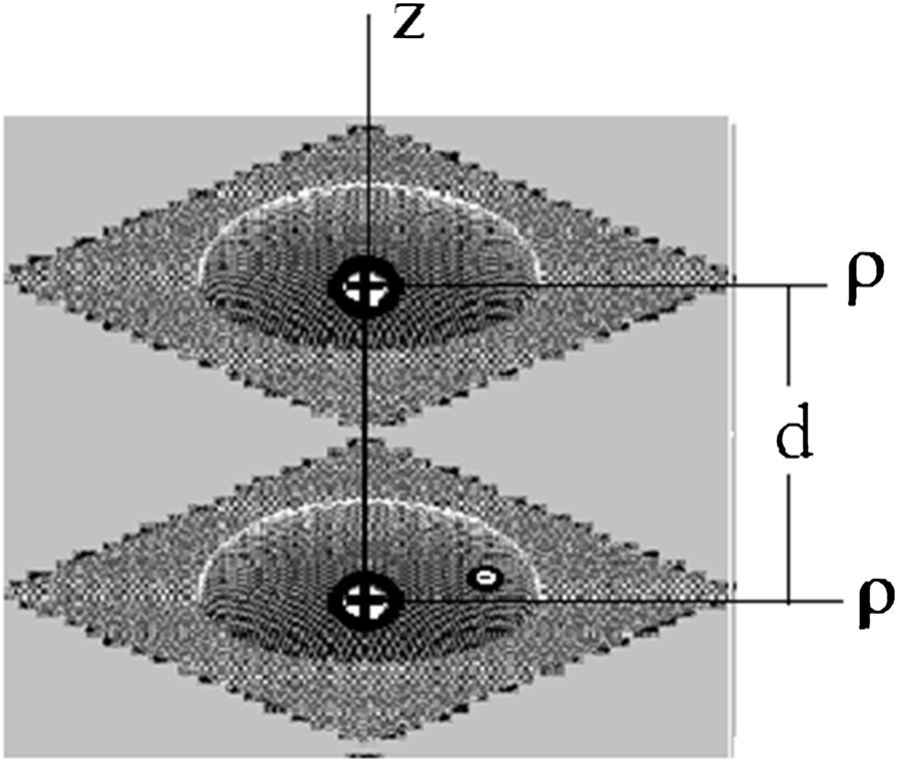
Image of the singly ionized molecule confined in lens-like QDs.

Below, we analyze the model of two separated on-axis singly ionized donors, confined in two coaxial, vertically stacked QDs, whose identical morphologies present axially symmetrical layers whose shape is given by the dependence of the layer thickness *h* on the distance *ρ* from the axis as follows: *h*(*ρ*) = *d*_*b*_ + *d*_0_*f*_*n*_(*ρ*)*ϑ*(*R*_0_ − *ρ*). Here, *R*_0_ is the base radius, *d*_*b*_ is the wetting layer thickness, *d*_0_ is the maximum height of the QD over this layer, *ϑ*(*x*) is the Heaviside step function, equal to 0 for *x* < 0 and to 1 for *x* > 0_,_ and *f*_*n*_(*ρ*) = [1 − (*ρ*/*R*_0_)^*n*^]^1/*n*^. The morphology is controlled in this model by means of the integer shape-generating parameter *n* which is equal to 1, 2, or tends to infinity for conical pyramid-like, lens-like, and disk-like geometrical shapes, respectively. As an example, the 3D image of an artificial singly ionized molecule confined in lens-like QDs is presented in Figure 1.

Besides, we assume that the external homogeneous magnetic field **B** = *Bẑ* is applied along the quantum dot's axis. The dimensionless Hamiltonian of the single electron in this *D*_2_^+^ complex in the effective-mass approximation can be written as

(1) $$H=-\Delta +V_{c}\left(\rho ,z\right)-\frac{2}{\left|r-R_{1}\right|}-\frac{2}{\left|r-R_{2}\right|};R_{i}=\left(0,z_{i},0\right)$$

where *V*_*c*_(*ρ*, *z*) is the confinement potential, equal to 0 and *V*_0_ inside and outside the QD, respectively. The last two terms in Equation 1 correspond to the attraction between electron and ions. The effective Bohr radius *a*_0_^*^ = ℏ^2^*ε*/m^*^e^2^, the effective Rydberg *R*_y_^*^ = *e*^2^/2*εa*_0_^*^, and *γ* = *e*ℏ*B*/2m^*^cR_y_^*^ have been taken above as units of length, energy, and the conventional dimensionless magnetic field strength, respectively.

As both donors are located at the axis, the potential is axially symmetrical, the angular momentum *L*_*z*_ commutes with the Hamiltonian, and the corresponding eigenvalues give us one good quantum number *m.* At this representation, the Hamiltonian (Equation 1) cylindrically coordinates only on two coordinates:

(2) $$\begin{array}{l} H_{m}\left(\rho ,z\right)=-\frac{1}{\rho }\frac{\partial }{\partial \rho }\rho \frac{\partial }{\partial \rho }-\frac{\partial^{2}}{\partial z^{2}}+\gamma m+\frac{\gamma^{2}\rho^{2}}{4}+V_{\rho }\left(\rho ,z\right);\\ V_{\rho }\left(\rho ,z\right)=V_{c}\left(\rho ,z\right)-\frac{2}{\sqrt{\rho^{2}+\left(z-Z_{1}^{2}\right)}}-\frac{2}{\sqrt{\rho^{2}+\left(z-Z_{2}^{2}\right)}}\text{.} \end{array}$$

Taking into account that the thickness of QDs is typically much smaller than their lateral dimension and therefore the electron motion in the first direction is much faster than in-plane motion, one can use the advantage of the adiabatic approximation [13] in which the wave function is presented as a product of two functions:

(3) $$\Psi_{m}\left(\rho ,z\right)=f\left(\rho ,z\right)\Phi_{m}\left(\rho \right);m=0,\pm 1,\pm 2,\ldots \text{,}$$

where the first function *f*(*ρ*, *z*) describes the fast motion in *z* direction and satisfies the wave equation

(4) $$-\frac{\partial^{2}f\left(\rho ,z\right)}{\partial z^{2}}+V\left(\rho ,z\right)f\left(\rho ,z\right)=E_{f}\left(\rho \right)f\left(\rho ,z\right)$$

with ‘frozen out’ radial coordinate *ρ*, while the radial part of the wave function is found in the second step from the equation

(5) $$\frac{1}{\rho }\frac{\partial }{\partial \rho }\rho \frac{\partial \Phi_{m}\left(\rho \right)}{\partial \rho }+\left[\gamma m+\frac{m^{2}}{\rho^{2}}+\frac{\gamma^{2}\rho^{2}}{4}+E_{f}\left(\rho \right)\right]\Phi_{m}\left(\rho \right)=E_{m}\Phi_{m}\left(\rho \right)\text{.}$$

In our numerical procedure, we solve Equation 4 repeatedly for each value *ρ* by using the trigonometric sweep method [13] in order to restore the unknown function *E*_*f*_(*ρ*). Once this function is found, then the energies *E*_*m*_ of the molecular complex can be established by solving Equation 5.

As the potential *V*(*ρ*, *z*) for each fixed value of *ρ* presents an even function *V*(*ρ*, − *z*) = *V*(*ρ*, *z*) with respect to the variable *z* corresponding to a symmetrical (no-rectangle) quantum well, then all solutions of Equation 4 can be arranged in two sets: odd solutions *f*^−^(*ρ*, − *z*) = − *f*^−^(*ρ*, *z*) and even solutions *f*^+^(*ρ*, − *z*) = *f*^+^(*ρ*, *z*), called antibonding and bonding states, respectively. These sets of functions can be found as the solutions of the boundary value problems corresponding to the differential Equation 4 within the range 0 <*z* < ∞ with the frontier conditions $\frac{d\;f^{+}\left(\rho ,0\right)}{dz}=0;f^{-}\left(\rho ,0\right)=0$.

## Results and discussion

We have performed numerical calculations of two-electron renormalized energies *E*_*m*_ as a function of the magnetic flux and for QDs with different morphologies, dimensions, and separation between layers in order to analyze the Aharonov-Bohm and the quantum size effects. We consider for our simulations the In_0.55_Al_0.45_As/Al_0.35_ Ga_0.65_As structures with the following values of physical parameters: dielectric constant *ε* = 12.71, the effective mass in the dot region and the region outside the dot for the electron *m* * = 0.076*m*_0_, the conduction and the valence band offset in junctions is *V*_0_ = 358meV, the effective Bohr radius *a*_0_^*^ ≈ 10nm, and the effective Rydberg Ry^*^ ≈ 5meV.

**Figure 2:**
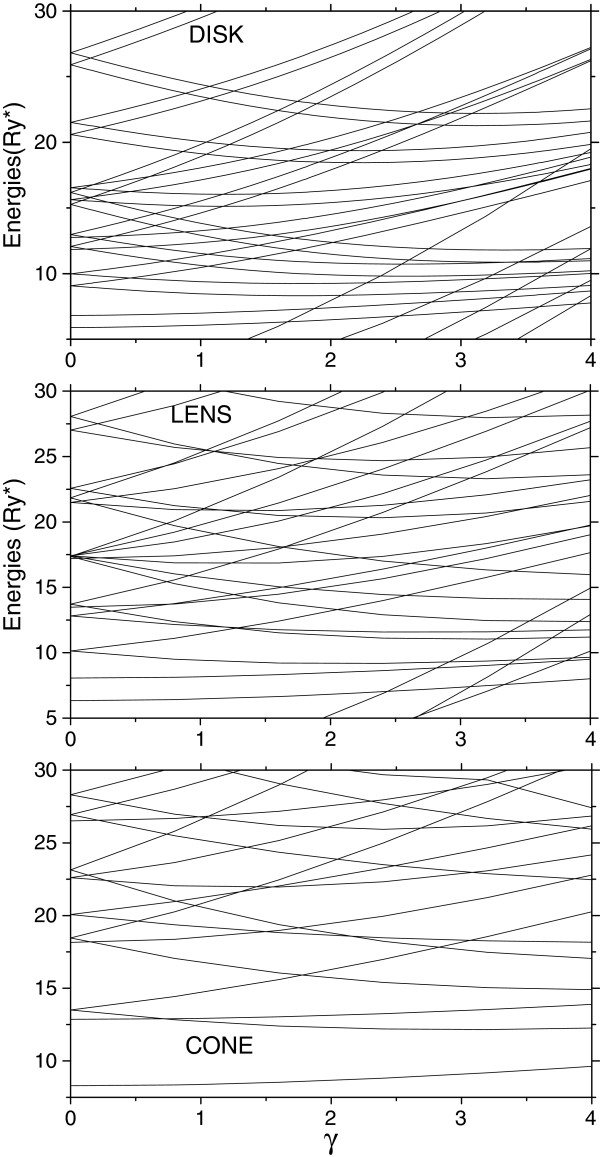
**Energies as functions of the magnetic field of a *D***_**2**_^**+ **^**in vertically coupled quantum dots.** (Heights 4 nm, wetting layer thicknesses 1 nm, radii 20 nm, and separation between them 6 nm).

First, we calculate the energies of the molecular complex as functions of the magnetic field in disk-like, lens-like, and cone-like vertically coupled QDs and in a single one-electron QR with smooth non-homogeneity of the surface. Results for vertically coupled QDs with the heights *d*_0_ = 4nm, the wetting layer thicknesses *d*_*b*_ = 1nm, radii *R*_0_ = 20nm, and the separation between them *d* = 6nm are shown in Figure 2.

It is seen that in all cases, the energy levels are very sensitive to the magnetic field and their dependencies on the magnetic field strength exhibit multiple crossovers and reordering. Comparing these dependencies for the disk, the lens, and the cone in Figure 2, one can also observe a successive increase of the number of crossovers and the lowering of the region energies where such crossovers occur. It is related to the variation of the electron probability distribution inside and around their InAs layers, which is similar to charge distribution in a metallic surface when its geometry varies from the flat to the spiked-type one. Such variation of the probability distribution is a consequence of the stronger confinement in structures with spiked-type QD geometry where the electron-ion separation is defined by interplays between the electrostatic interaction between them and the strong structural confinement, making it more stable with respect to the external magnetic field and the ring-like electron probability density distribution. Therefore, the energy dependencies for cone-like QDs have a shape similar to those that exhibit structures with ring-like geometry known as the Aharonov-Bohm effect.

The Aharonov-Bohm effect observed usually in ring-like heterostructures is a manifestation of the competition between the paramagnetic and diamagnetic terms in the Hamiltonian, resulting in the oscillation of the ground state energy. Such oscillations are impossible in the disk-like structures because of a significant decrease of the diamagnetic term contribution as the magnetic field increases and the electron probability distribution becomes more contracted. In QDs with a spike-like morphology, the electron probability density is already strongly confined, the external magnetic field can no longer decrease more the diamagnetic term contribution, and the energy dependencies on the increasing magnetic field become similar to those of ring-like structures.

**Figure 3:**
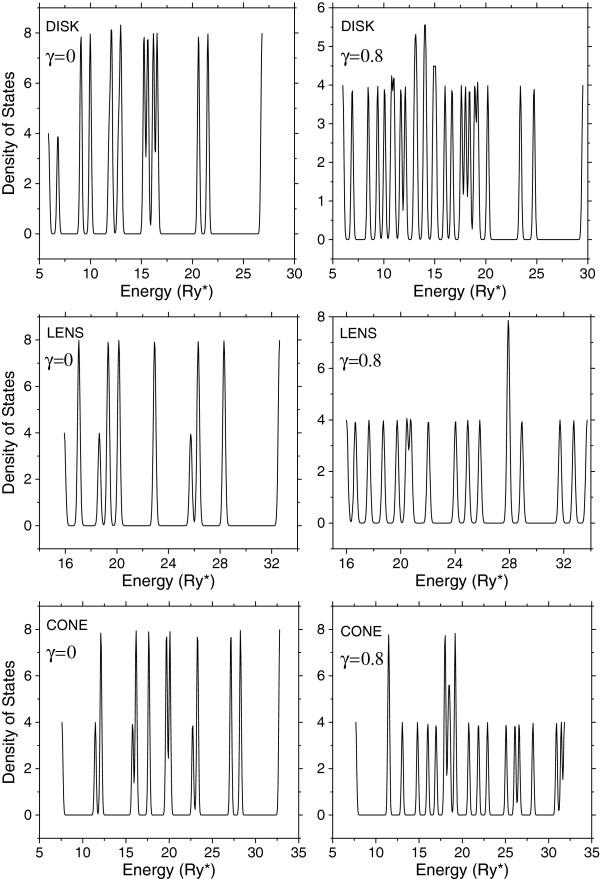
**Density of the electronic states for a *D***_**2**_^**+ **^**in vertically coupled quantum dots.** (Heights 3 nm, wetting layer thicknesses 2 nm, radii 20 nm, and separation between them 6 nm for two different values of the magnetic field (*γ* = 0) and (*γ* = 0.8)).

In Figure 3, we present results of the calculation of the density of electronic states in the zero-magnetic field for QDs with three different morphologies on the left side case *γ* = 0 and on the right side for *γ* = 0.8. It is seen that the density of electronic states in the case of the zero-magnetic field for the disk-like structure has a larger value in the region of the low-lying energy levels and it decreases successively while the morphology becomes more and more spike-liked. It is due to the fact that the electron confinement in the disk is weaker than that in the lens and that in the lens is weaker than that in the cone.

Also, it is seen that the lowest peak corresponding to the ground bonding state in the cone-like structure is more significantly separated from other excited states than in two other structures. It is due to the stronger confinement of the electron in the cone-like structure where the electron is mainly located nearer to the donor than in disk-like and lens-like structures.

Comparing the densities of states presented on the left and right sides of Figure 3, one can see remarkable modifications that suffer the corresponding curves. Particularly, in the disk-like structure, the presence of the magnetic field provides a displacement of the peaks at the region of the low-lying energies. In the lens-like and cone-like structures, the modification is inversed; the peaks are reorganized in such a way that their distribution becomes almost homogeneous. Redistribution of the peaks' positions in the lens is defined mainly by the additional confinement that provides the external magnetic field, while analogous redistribution in other two spike-liked structures is mainly due to the Aharonov-Bohm effect.

## Conclusions

In short, we propose a simple numerical procedure for calculating the energies and wave functions of a singly ionized molecular complex formed by two separated on-axis donors located at vertically coupled QDs in the presence of the external magnetic field. Our calculation includes some important characteristics of the heterostructure such as the presence of the wetting layer and the possibility of the variation of the QD morphology. The curves of the energy dependencies on the external magnetic field for the disk-like, lens-like, and cone-like structures are presented. We find that the effect of the in-plane confinement on the electron-ion separation is stronger in spike-shaped QDs and therefore the energy dependencies in such structures exhibit a behavior similar to that in ring-like structures. The analysis of the curves of the density of electronic states also confirms this result.

## Competing interests

The authors declare that they have no competing interests.

## Authors’ contributions

All authors contributed equally to this work. JSO created the analytic model with contributions from IM. RMG and GES performed the numerical calculations and wrote the manuscript. All authors discussed the results and implications and commented on the manuscript at all stages. All authors read and approved the final manuscript.

## Authors’ information

JSO obtained his Ph.D. in 2004 at the Universidad Industrial de Santander, where IM was his advisor. His research interests include the theory of semiconductor nanostructures. JSO is the head of the research group ‘Condensed Matter Theory’ at the University of Magdalena. GES and RMG are master's degree and Ph.D. students, respectively, and teachers at the University of Magdalena.
